# Impact of SARS-CoV-2 P.1 Variant Infection on the Nasopharyngeal Commensal Bacterial Microbiome of Individuals from the Brazilian Amazon

**DOI:** 10.3390/microorganisms13051088

**Published:** 2025-05-08

**Authors:** Amanda Mendes Silva Cruz, Jedson Ferreira Cardoso, Kenny Costa Pinheiro, Jessylene Almeida Ferreira, Luana Soares Barbagelata, Sandro Patroca Silva, Wanderley Dias Chagas Junior, Patrícia Santos Lobo, Dielle Monteiro Teixeira, Walter André Junior, Inaiah Ordenes Silva, Mirleide Cordeiro Santos, Luana Silva Soares Farias, Maisa Silva Sousa, Fernando Neto Tavares

**Affiliations:** 1 Virology Section, Evandro Chagas Institute, Secretariat for Health and Environmental Surveillance, Ministry of Health, Highway BR 316-KM 07, S/N, Levilândia, Ananindeua 67030-000, PA, Brazil; amandamendes@iec.gov.br (A.M.S.C.); jedson.cardoso@gmail.com (J.F.C.); kennybiotec@gmail.com (K.C.P.); jessylene_almeida@hotmail.com (J.A.F.); luanabarbagelata@iec.gov.br (L.S.B.); wanderleychagas@iec.gov.br (W.D.C.J.); patricialobo@iec.gov.br (P.S.L.); dielleteixeira@iec.gov.br (D.M.T.); mirleidesantos@iec.gov.br (M.C.S.); luanasoares@iec.gov.br (L.S.S.F.); fernandotavares@iec.gov.br (F.N.T.); 2 Center for Tropical Medicine, Federal University of Pará, Av. Generalíssimo Deodoro, 92, Umarizal, Belém 66055-240, PA, Brazil; 3 Arbovirology Section, Evandro Chagas Institute, Secretariat for Health and Environmental Surveillance, Ministry of Health, Highway BR 316-KM 07, S/N, Levilândia, Ananindeua 67030-000, PA, Brazil; spatroca@gmail.com; 4 Ministry of Health, Central Public Health Laboratory of Amazonas, R. Emílio Moreira, 528—Centro, Manaus 69020-040, AM, Brazil; walter.andre.jr@hotmail.com; 5 Ministry of Health, Amazonas Health Surveillance Foundation-Dr. Rosemary Costa, Torquato Tapajós Avenue, 4010—Santo Antônio College, Manaus 69093-018, AM, Brazil; inaiah.ordones@gmail.com

**Keywords:** SARS-CoV-2, P.1 variant, nasopharyngeal, alpha diversity, beta diversity, metagenomic, Brazilian Amazon

## Abstract

It is important to understand which bacterial taxa are most abundant during SARS-CoV-2 infection and to promote mitigation strategies for conditions subsequent to infection. Nasopharyngeal swab samples were collected from patients infected with SARS-CoV-2 and their family contacts (uninfected and asymptomatic) during the outbreak of the P.1 variant of SARS-CoV-2 in Parintins, Amazonas–Brazil, in March 2021. The samples were investigated by a shotgun sequencing metagenomic approach using the NextSeq 500 Illumina® system. The samples were stratified according to the presence or absence of SARS-CoV-2, household group, sex, and age. Of the total of 63 individuals, 37 (58.73%) were positive for SARS-CoV-2 and 26 (41.27%) were negative for SARS-CoV-2 and other respiratory viruses (FLU, AdV, HBoV, HCoV, HMPV, RSV, PIV, HRV). The alpha diversity indexes Chao1, species observed, Simpson, and Inv Simpson demonstrated a significant difference (*p* < 0.05) in both the diversity of observed species and the abundance of some taxa between positive and negative individuals. We also observed an abundance of opportunists such as *Klebsiella pneumoniae*, *Staphylococcus* spp, and *Shigella sonnei*, previously associated with the severity of COVID-19. Our results suggest that SARS-CoV-2 infection causes changes in the microenvironment of the nasopharyngeal region, allowing greater proliferation of opportunistic bacteria and decreased abundance of commensal bacteria.

## 1. Introduction

Immediately after the emergence of Severe Acute Respiratory Syndrome Coronavirus 2 (SARS-CoV-2) and subsequent coronavirus disease pandemic 2019 (COVID-19) [1], global scientific efforts have been directed towards understanding viral biology [2,3], epidemiology, and pathophysiology of these infections [4]. However, little is known about its effect on the microbiome of the upper respiratory tract (URT), leaving gaps in knowledge about the etiological agents secondary to SARS-CoV-2 infection, which may be playing some role in the clinical worsening of these patients [5].

In this system, the nasopharynx is an anatomically unique region, which serves as a connection point between the cavities of the ear, nose, and mouth [6,7]. This characteristic creates a dynamic and complex microenvironment that is colonized by a wide variety of bacteria [7,8]. These bacteria play a key role in maintaining the health of the upper respiratory tract, helping to protect against pathogenic invaders and facilitating immune function [9,10]. In addition, the nasopharynx exhibits a greater diversity of microbial communities than other parts of the URT [9,11,12].

In general, the mucosal surfaces of the nasopharynx of healthy individuals are colonized by commensal and opportunistic bacteria. The majority are members of the Firmicutes, Actinobacteria, Bacteroidetes, and Proteobacteria phyla [7,9,12,13]. However, large differences in microbial profiles can be observed between niches at lower taxonomic levels, especially when this environment is disturbed [9,14,15,16]. Therefore, any changes in the diversity and population of the microbiome lead to an interruption in homeostasis, causing a state of “dysbiosis”.

In this sense, it is known that respiratory viruses alter the composition of the microbial community of the respiratory tract, resulting in unevenness (upwards or downwards) of commensal microbial diversity and greater abundance of opportunistic pathogens that promote the severity of the disease in infected individuals [17,18,19]. In addition, secondary infection or co-infection with bacteria is common and usually progressive [8,20]. However, different viruses interact with different bacterial groups, making the dynamics of interaction and their outcomes even more complicated [9]. In view of the above, growing evidence from metagenomic approaches indicates that SARS-CoV-2 infection has a significant effect on the diversity and composition of the bacterial community in the nasopharynx of humans [15,16,21,22]. The presence of secondary etiological agents in SARS-CoV-2 infections has significant clinical relevance, as it can contribute to respiratory complications and worsening of the clinical picture [15,16].

In this scenario, the identification and monitoring of these microorganisms are essential for the appropriate choice of antimicrobial therapies and intensive care. Therefore, the clinical importance of investigating and understanding the role of these secondary agents in the context of COVID-19 is indisputable in order to improve clinical outcomes and patient survival. To this end, describing the differences in taxon abundance in microbiomes of infected and uninfected patients can provide information that may shed light on the conditions following SARS-CoV-2 infection. Therefore, this study aimed to describe the differences in the nasopharyngeal microbiome of a retrospective cohort of uninfected and infected individuals with SARS-CoV-2 in a population located in the city of Parintins, Amazonas, Brazil, in 2021.

## 2. Materials and Methods

### 2.1. Ethical Statement

This study was approved by the Human Research Ethics Committee of the Evandro Chagas Institute, protocol number 79631424.6.0000.0019, in accordance with Resolution 466/2012 of the National Health Council. The authors assure that all procedures contributing to this work comply with the ethical standards of the relevant national and institutional committees on human experimentation and with the Declaration of Helsinki of 1975, as revised in 2008.

### 2.2. Study Population and Sample Collection

In March 2021, the positive case tracking database of the Health Surveillance Foundation and the Municipal Health Department of Parintins, Amazonas, Brazil, was used to select individuals with a positive laboratory diagnosis for SARS-CoV-2 in the last three days. The individuals were treated by the medical team of Municipal Health Units and nasopharyngeal samples were collected by the Respiratory Syndrome Surveillance team. For collection, rayon swabs and Viral Transport Medium (VTM) containing antibacterials and antifungals provided by the Ministry of Health were used. The samples were stored at a temperature of 8 °C and immediately sent to the Central Laboratory of the State of Amazonas to proceed with molecular diagnosis (RT-qPCR). After diagnosis, samples were stored at −70 °C. Individuals who tested positive for SARS-CoV-2 received a home visit from the Health Surveillance technical team (composed of a nurse and an epidemiologist) to collect nasopharyngeal swabs from their family members, following the previously reported flow. After analysis by RT-qPCR (Allplex 2019-nCov Assay Kit), samples were stored at −70 °C and sent on dry ice to the Respiratory Virus Laboratory of the Evandro Chagas Institute, Ananindeua, Pará, Brazil, for further analysis. Transportation time was up to 24 h.

### 2.3. Tests to Detect the Genome of SARS-CoV-2 and Other Respiratory Viruses

Total nucleic acid was used for RT-qPCR molecular diagnostics with the GoTaq® Probe 1-Step RT-qPCR System kit (PROMEGA, Madison, WI, USA). Specific primers and probes were used for SARS-CoV-2, influenza A virus (FluA), influenza B virus (FluB), adenovirus (AdV), human bocavirus (HBoV), human coronavirus (HCoV) 229E, HCoV-HKU1, HCoV-NL63, and HCoV-OC43, human metapenumovirus (HMPV), Human parainfluenza virus (HPIV 1-3), Respiratory Syncytial Virus (RSV) and human rhinovirus (HRV), according to the protocol standardized by the CDC [23]. At the end of the reaction, samples were considered positive up to a 40 cycles threshold. Positive and negative controls were included in each reaction.

### 2.4. Shotgun Metagenomic Next-Generation Sequencing

Total nucleic acid was quantified using the Qubit™ dsDNA HS Assay Kit (Invitrogen) and samples reached a concentration equal to or greater than 1 ng/µL went to the next stage. The DNA libraries were prepared using the Nextera XT DNA Library Prep Kit (Illumina®, San Diego, CA, USA) and the Agilent 2200 TapeStation system (Agilent Technologies–Waldbronn, Germany) plus the High-Sensitivity D1000 Reagents and High-Sensitivity D1000 ScrenTape kit was used as control of quality for the fragments generated during library prepararation. Sequencing was carried out using NextSeq Illumina® sequencing systems. A total of 800 million paired-end reads were available for every 20 samples, ensuring approximately 40 million reads per sample.

### 2.5. Bioinformatics Analysis

The quality control of the raw data (reads) in FASTQ format was carried out using the set of tools implemented in KneadData v.0.6.1. Initially, FASTQC v.0.12.0 [24] was used to evaluate the number of bases sequenced and compare the quality of the reads before and after the quality filter, thus guiding the choice of the cut-off value (PHRED ≥ 20). Trimming was carried out using Trimmomatic v.0.36 [25], removing low-quality readings and possible adapters. Tandem repeats in the DNA of the sequenced reads were also removed using the Tandem Repeat Finder (TRF) tool. Next, reads mapped to the human reference genome GRCh38 were discarded using Bowtie2 v.2.4.4 [26]. Only paired reads that passed the filtering were kept, and these were classified as non-host reads. In order to elucidate the microbiome of SARS-CoV-2 positive and negative patients, the readings after all previous filtering phases were compared using BlastX implemented in Diamond v.2.0.11.149 [27] against the NR Database (NCBI’s nonredundant protein database), where the statistical value, e-value [28], considered was 1.0 × 10 ^−4^, and the BlastX results were plotted using the KRONA v.2.7 software [29]. The program MEGAN v.6.24.20 [30] was used to perform taxonomic and functional binning of the sequences based on the resulting Diamond alignments (DAA format), where the statistical value, e-value [28], considered was 1.0 and −11; such values consider a high probability of taxonomic homology assigned to the respective reads when compared to the NR database.

### 2.6. Statistical Analysis

The taxonomic classification data generated by the MEGAN v.6.25.10. software were converted into BIOM format and imported into the R statistical software environment, version 4.1.3. [31], using the phyloseq package [32]. Statistical inferences were made using the R software [27] and a *p*-value of less than 0.05 (*p*-value < 0.05) was considered statistically significant. The alpha diversity calculations were based on the taxonomic analysis of abundance at the family and species level and the results were visualized in boxplots. The alpha diversity indices used were species observed, Chao1, InvSimpson, Simpson, and Shannon; they were calculated in R using the phyloseq package [32] using the plot_richness function. Wilcoxon rank sum tests were performed between detectable and nondetectable groups for SARS-CoV-2, between groups separated by gender and groups separated by age, to determine the differential abundance of the metagenomic characteristics associated with these samples. Wilcoxon tests were also carried out for different members of family groups. Principal co-ordinate analyses (PCoA) of beta diversity were calculated from the taxonomic analysis of relative abundance at the family and species level. Bray–Curtis diversity was calculated in R using the R package phyloseq [32] using the ordinate function. Bar graphs of absolute frequency at the family and species level were generated from the results of the taxonomic analysis produced by the MEGAN software [30]. The bar charts were separated into categories based on the presence or absence of SARS-CoV-2 and the results of the frequency of taxa observed in these categories were visualized using the ggplot package2 [33] package in the R statistical environment. Venn plots were also produced in order to count the number of taxa identified only in SARS-CoV-2 positive and negative samples, as well as the number of taxa shared between the two categories. For the analyses of median and interquartile range of the age, the Lilliefors test and *t*-test were used.

## 3. Results

### 3.1. Sample Characteristics and Study Population

RT-qPCR diagnosis of 63 nasopharyngeal samples tested identified 26 negative and 37 positive samples for SARS-CoV-2. To rule out the possibility of infection by other respiratory viruses, the samples (positive and negative) were differentially diagnosed for influenza A and B viruses, human respiratory syncytial virus, metapneumovirus, adenovirus, rhinovirus, parainfluenza 1, 2, and 3, bocavirus, and the coronaviruses OC43, HKU1, NL63, and 229E. The genome of none of the 14 respiratory viruses investigated was detected. The samples were categorized according to the presence or absence of SARS-CoV-2, family group, gender, and age. The ages of positive and negative cases presented a normal distribution. The age of negative cases ranges from 19 to 72 years, with a mean of 32.7 years (sd ± 13.3). The positive cases had an age range from 20 to 75 years, with a mean of 40.9 years (sd ± 14.7). The mean age of positive cases was significantly higher than that of negative cases (*p* = 0.0263). The age of positive men ranges from 20 to 75 years, with a mean of 38.8 years (sd ± 13.3). Among positive women, the age ranges from 21 to 73 years, with a mean of 43.4 years (sd ± 16.4). There was no significant difference between the ages of positive men and women (*p* = 0.3501).

A summary of the categories is shown in Table 1. Detailed information on the samples is described in Supplementary Material S1.

### 3.2. Microbial Diversity of Individuals Infected and Not Infected with SARS-CoV-2

**Figure 1:**
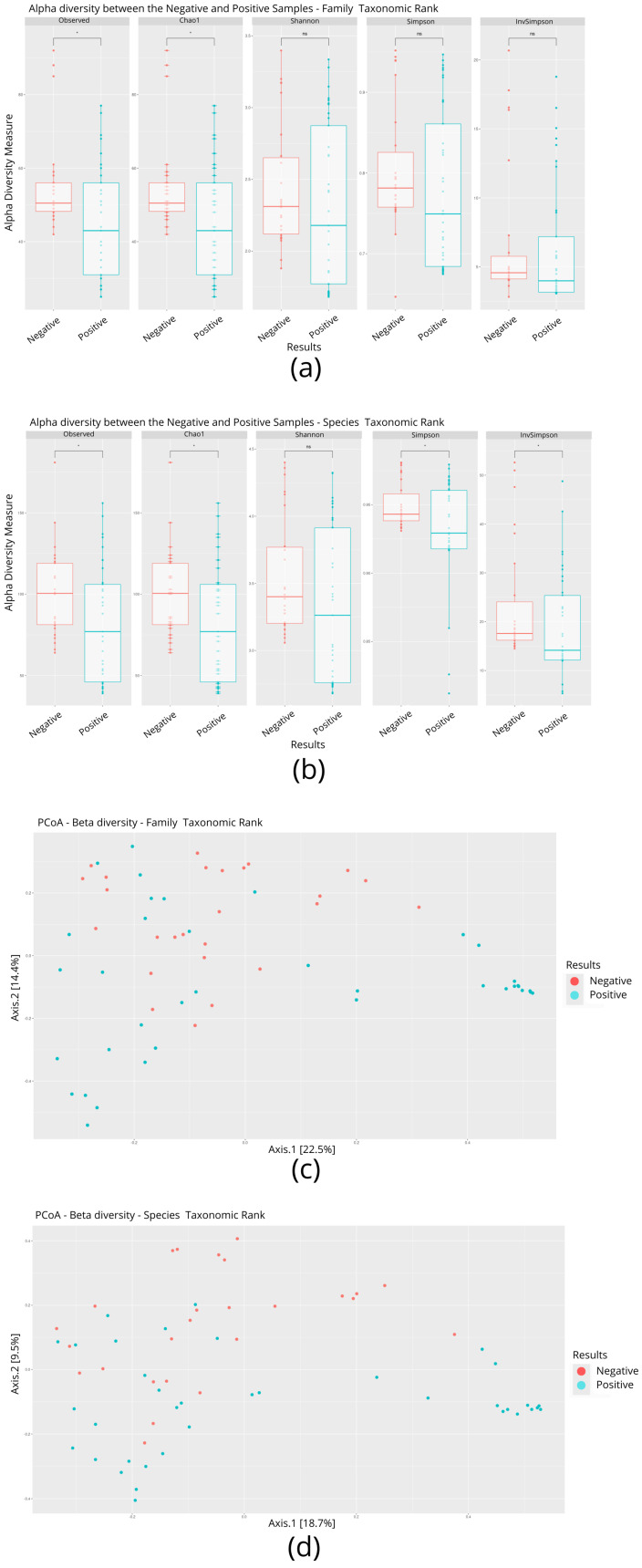
Comparison of alpha and beta diversity parameters between individuals infected and not infected with SARS-CoV-2. (**a**) Boxplot graphs of alpha diversity indices at the family level. (**b**) Boxplot graphs of alpha diversity indices at the species level. A *p*-value < 0.05 is represented by an asterisk (*). (**c**) Analysis of beta diversity at the family level. (**d**) Analysis of beta diversity at the species level.

The nasal microbial profile of individuals infected (*n* = 37) and not infected by SARS-CoV-2 (*n* = 26) was determined by shotgun metagenomic sequencing. Each metagenome was recovered, resulting in approximately 42,705,418 million reads obtained after the quality filter. The readings corresponding to everyone individuals, were analyzed for the taxonomic inferences of alpha and beta diversity after filtering and, for alpha diversity at the family level, statistical parameters such as the Shannon, Simpson, and InvSimpson indices showed no significant differences (*p* < 0.05) between individuals infected and not infected by SARS-CoV-2 (Figure 1a). In contrast, the values observed in the observed and Chao1 statistical parameters were significantly (*p* < 0.05) lower in infected individuals when compared to uninfected individuals (Figure 1a). At the species level, four of the five parameters analyzed (observed, Chao1, Simpson, and InvSimpson) showed a significant difference between individuals infected and not infected by SARS-CoV-2 (Figure 1b). The beta diversity statistical parameters such as principal co-ordinates analysis (PCoA) showed no significant differences between the two groups, either at the family or species level (Figure 1c,d).

**Figure 2:**
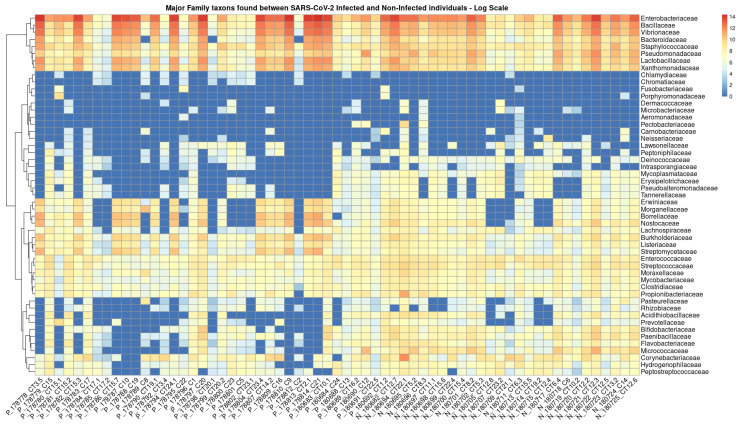
Heat map based on the numbers of reads of the main taxa that have changed in abundance between individuals infected (prefix P) and not infected (prefix N) by SARS-CoV-2. The clinical samples are listed in the bottom text line and the names of the bacterial families are shown in the text column on the left. The blue to red colored boxes represents the observed metagenomic sequencing reads (reads ranged from 0 to 14 on the logarithmic scale, respectively).

**Figure 3:**
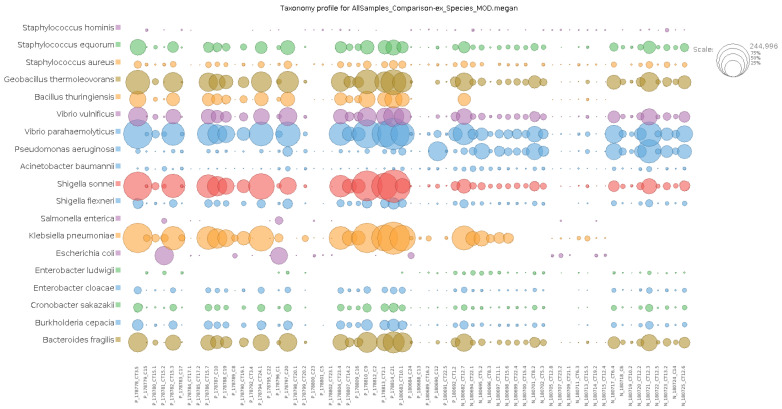
Comparative analysis of the most abundant taxa at species level of the infected and uninfected with SARS-CoV-2. Note: the prefixes P and N denote infected and non-infected, respectively.

**Figure 4:**
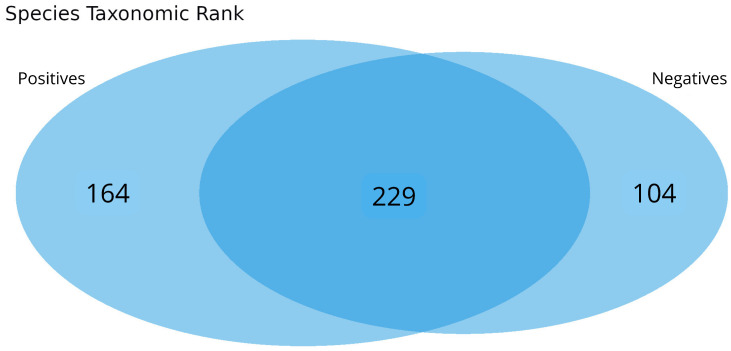
Taxonomic composition of the nasopharyngeal microbiomes of individuals infected or not with SARS-CoV-2. The Venn diagram shows unique and shared bacterial species between the groups.

In total, 136 distinct bacterial taxa at the family level were observed with at least 16 families in each nasopharyngeal sample (Supplementary Material S2). Most of the taxa (64.70%) were read at least 1000 times and 50 families obtained more than 10,000 readings each. Among the taxa with the highest number of reads (≥1,000,000 reads), the families *Enterobacteriaceae* (20,678,814 reads ~48%), *Bacillaceae* (5,793,607 reads ~13%), *Vibrionaceae* (4,075,955 reads ~9%), *Lactobacillaceae* (1,778,346 reads ~4%), *Bacteroidaceae* (1,286,215 reads ~2%), *Staphylococcaceae* (1,269,907 reads ~2%), *Xanthomonadaceae*, 1,109,975 reads ~2%), and *Pseudomonadaceae* (1,039,830 reads ~2%) made up the majority of the nasal microbiome nasopharyngeal of infected and uninfected individuals (Figure 2). At the species level, the abundance of *Staphylococcus equorun*, *Geobacillius thermoleovorans*, *Bacilus thuringiensis*, *Shigella sonnei,* and *Klebsiella pneumoniae* was significantly higher in individuals infected with SARS-CoV-2 (Figure 3). In contrast, *Pseudomonas aeruginosa* was considerably increased uninfected individuals (Figure 3). The unique and shared distribution of bacteria found in the two groups of participants is represented by Venn diagram, as shown in Figure 4. In this study, 497 bacterial species were detected, of which 393 and 333 were reported in infected and uninfected individuals, respectively, and 229 (46.07%) species were common in both groups of individuals (Figure 4).

### 3.3. Intra-Family and Inter-Family Microbial Profile

**Figure 5:**
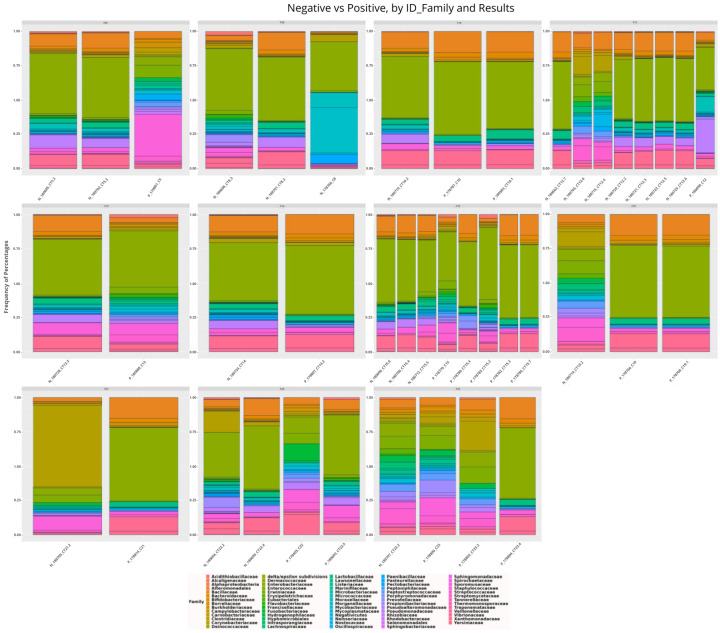
Taxonomic distribution of the main bacterial families among members of 11 different households of individuals infected and not infected with SARS-CoV-2. Note: prefixes F denote different house-holds.

In order to describe the nasopharyngeal microbiome of members of the same household and compare it with that of other households, only households with at least one infected member and one uninfected member made up this analysis. Thus, of the total microbiomes analyzed, 42 belonged to individuals from 11 different households. It was observed that the composition of the bacterial community of the nasopharynx of the members of these households is similar, with a slight variation in abundance (Figure 5). In most cases, the greater the bacterial diversity, the lower the abundance of taxa in infected individuals.

### 3.4. Bacterial Communities Associated with Age Groups and Sex

**Figure 6:**
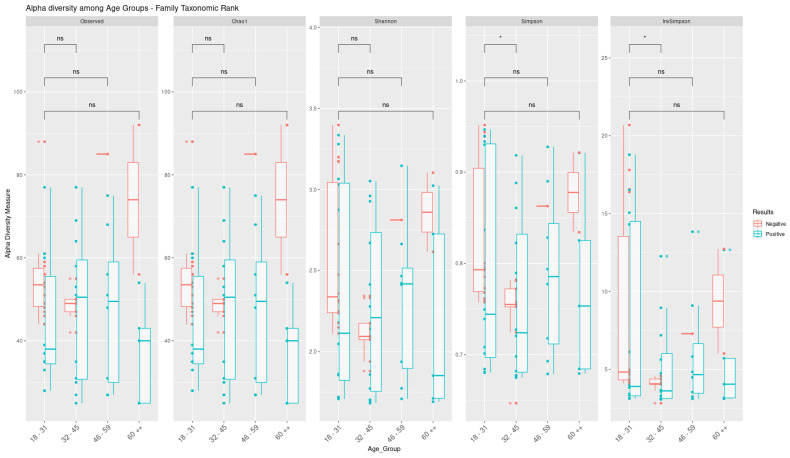
Comparison of alpha diversity parameters at family level among individuals infected with SARS-CoV-2 stratified by age group. Box–whisker plots of the alpha diversity indices and their comparison were generated from the results of the Wilcoxon test. The asterisk symbol (*) indicates significant (*p* < 0.05) and symbol (ns) indicates not significant.

Alpha diversity was assessed for four age groups (age group 1: 18–31 years; age group 2: 32–45 years; age group 3: 46–59 years; and group 4: ≥60 years) and by gender (male: n = 29, 9 uninfected and 20 infected; female: n = 34, 17 uninfected and 17 infected) at both the family and species level. Figure 6 shows the results of alpha diversity at the taxonomic family level in terms of the absence or presence of SARS-CoV-2. A statistically significant difference (*p* < 0.05) was observed according to the nonparametric Wilcoxon test between age groups 1 and 2 in the Simpson and InvSimpson indices, and, when comparing the distribution graphs (boxplots) between positive and negative individuals, it was possible to identify a downward trend in microbial diversity for age group 2 (32–45 years).

**Figure 7:**
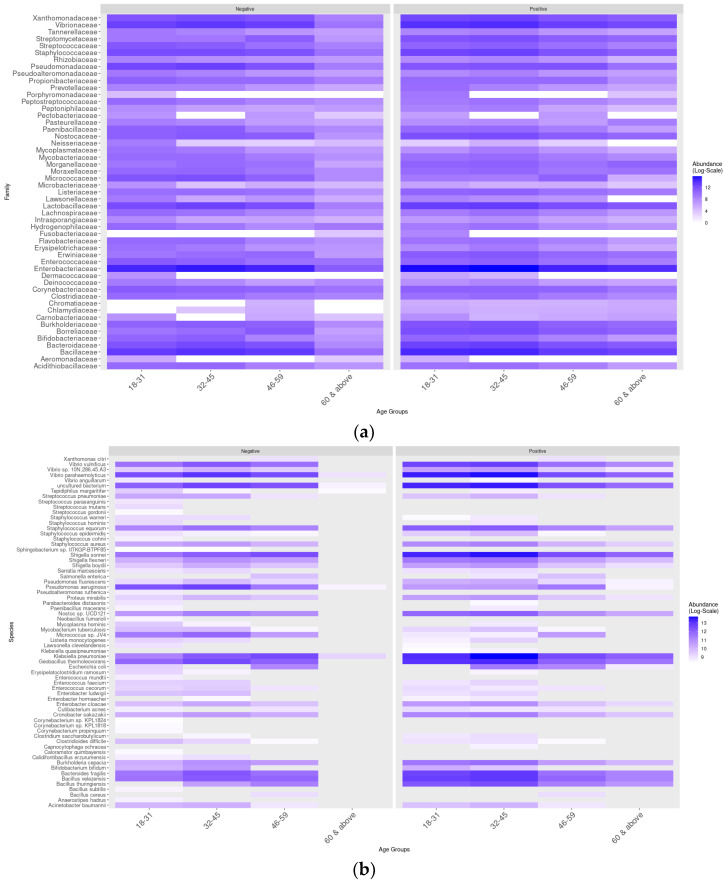
Taxonomic distribution of the main bacterial families and species according to age group and sex. (**a**) Distribution based on the heat map of the main taxa in different age groups. (**b**) Distribution based on the heat map of the main taxa in different age groups.

The distribution of the main taxa based on heat maps exposed a difference in their pattern of abundance (Figure 7). In age group 1 (18–31 years), the bacterial families *Tannerellaceae*, *Streptococcaceae*, *Nostococcaceae*, and *Porphyromonadaceae* were more abundant in infected individuals compared to uninfected ones (Figure 7a). On the other hand, the species *Streptococcus parasanguinis*, *Streptococcus mutans,* and *Staphylococcus hominis* were only present in uninfected individuals, and, curiously, *Escherichia coli* was not observed (Figure 7b). In contrast, *Shigella sonnei*, *Klebsiella pneumoniae*, *Geobacillus thermoleovorans,* and *Vibrio parahemolyticus* were enriched in the microbiomes of infected individuals (Figure 7b). In age group 2 (32–45 years), *Shigella sonnei* and *Klebsiella pneumoniae* were more abundant in the microbiomes of infected individuals, while *Pseudomonas aeruginosa* was less abundant. In age group 3 (46–59 years), the distribution of abundance and taxonomic diversity was similar between the groups of infected and uninfected individuals. In contrast, in age group 4, these parameters were markedly increased in infected individuals. In general, it was shown that some taxa were less abundant in age groups 3 and 4 compared to age groups 1 and 2, and *Klebsiella pneumoniae* was significantly more abundant in age group 2 compared to the others.

**Figure 8:**
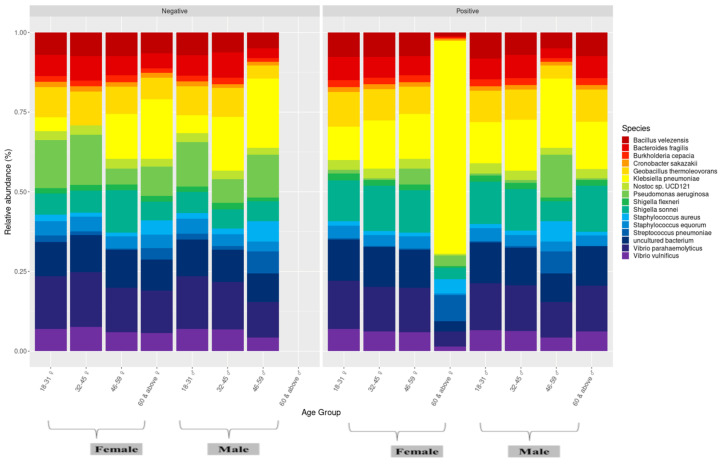
Microbiome profile of male and female individuals between age groups.

We also compared the nasopharyngeal microbiome of men and women infected and uninfected by SARS-CoV-2 between age groups. It was observed that Pseudomonas aeruginosa was more abundant among those not infected by SARS-CoV-2 and that *Bacillus thuringiensis* was more abundant among those infected by SARS-CoV-2 regardless of sex and age group. Among the younger population (18–31 years), no significant differences were observed regardless of sex. For women aged 32 to 45 years, *Klebsiella pneumoniae* was observed only in those infected by SARS-CoV-2. In the elderly infected >60 years, *Klebsiella pneumoniae* and *Staphylococcus equorum* were more abundant in women and *Bacillus velezensis*, *Cronobacter sakazakii,* and *Bacteroides fragilis* were more abundant in men in this age group (Figure 8).

## 4. Discussion

To our knowledge, this is the first study that explicitly focused on the nasopharyngeal bacterial composition of uninfected and SARS-CoV-2-infected individuals from a population in the Amazon region where the P.1 variant emerged. In this study, we found dysbiosis in the nasopharynx of individuals infected with SARS-CoV-2 when compared to uninfected individuals (*p* < 0.05). Subgroup analyses showed that young adults infected with SARS-CoV-2 (age groups of 18–31 and 32–45) had a higher abundance of opportunistic pathogens. In addition, men and women had a similar bacterial community, as observed among members of the same household.

Our data suggest that SARS-CoV-2 infection favored the decrease in alpha diversity when evaluating the richness metric (*p* < 0.05: observed species, Chao 1, Simpson and InvSimpson), demonstrating a difference in diversity and abundance of the observed species between individuals infected and not infected by SARS-CoV-2. These results are in accordance with previously published studies [16,22,34,35,36,37].

It is worth noting that the nasopharyngeal bacterial microbiome of the individuals studied consists of at least 16 bacterial groups. However, the abundance of each taxon varied between the infected and uninfected groups. In infected patients, bacteria from the *Enterobacteriaceae*, *Bacillaceae*, *Vibrionaceae*, *Lactobacillaceae*, *Bacteroidaceae*, and *Staphylococcaceae* families were frequently detected, supporting the findings of previous studies [38,39,40].

Studies have reported the communication of the gut and nasal microbiome and that SARS-CoV-2 infection change the gut microbiome, allowing pathogenic bacteria such as Enterobacteriaceae to thrive and triggering more severe disease outcomes [39,41,42]. In addition, Gratiela Gradisteanu et al. (2023) explained that SARS-CoV-2 infection led to exacerbated changes in the microbiome in patients with type 2 diabetes, characterized by higher levels of Enterobacteriaceae [43].

Regarding the influence of *Bacteroidaceae* on the course of SARS-CoV-2 infection, Tao Zuo et al. (2020) reported that *Bacteroides* spp play an important role as a member of the gut microbiome against COVID-19 infection, negatively regulating angiotensin-converting enzyme 2 (ACE2) and reducing the entry of SARS-CoV-2 [39]. The genus *Bacteroide* is known to perform immunomodulatory actions in the human gastrointestinal system [44] and, consequently, potentially exerts the same action in the respiratory system. In this sense, previous studies have reported that these members constitute the central microbiome of the nasal community and harbor various opportunistic pathogens, which alter the host’s immune response during influenza infections, which may be occurring in SARS-CoV-2 infections [36].

Research shows that the abundance of *Bacillaceae* species may increase the inflammatory response and impact the severity of COVID-19 [40]. Conversely, Sadia Alam et al. (2020) proposed using compounds derived from *Bacillus* spp as potential inhibitors of key proteases in the coronavirus replication cycle [45]. Therefore, enriching this taxon could have an ambiguous effect, either contributing to the clinical condition of individuals or aiding in controlling the infection.

As researchers delved deeper into the taxonomic classification, we observed that *Klebsiella pneumoniae* and *Staphylococcus* spp were the most frequently identified pathogens in the nasopharynx of SARS-CoV-2 infected individuals. According to Sharifipour et al. (2020), in a study of COVID-19 patients, almost half (10 out of 21) of the patients developed secondary bacterial infections in the lungs caused by *Klebsiella pneumoniae* and *Staphylococcus* spp. Sadly, this ultimately resulted in death despite receiving antibiotic treatment [46]. Although, in the present study, these bacteria were expressively abundant in the nasopharyngeal microbiome, the communication of the entire respiratory tract allows the movement and increase in abundance of bacteria from one region to another [8].

We also draw attention to the significant number of readings associated with *Geobacillius thermoleovorans* in both groups (infected and uninfected), although they were more abundant in patients infected with SARS-CoV-2. It was reported that this bacterium was markedly increased in children with severe COVID-19 and Multisystem Inflammatory Syndrome [47]. However, a study on potential probiotic candidates revealed that *Geobacillius thermoleovorans* has an inhibitory effect against pathogenic bacteria, especially Gram-negative bacteria [48]. In our study, the abundance of *Geobacillius thermoleovorans* was concomitantly observed with a lower abundance of *Pseudomonas aeruginosa* and *Escherichia coli* among individuals infected with SARS-CoV-2. Interestingly, other authors demonstrated a higher abundance of *Pseudomonas aeruginosa* and *Escherichia coli* and the absence of *Geobacillius thermoleovorans* [49,50]. Therefore, we hypothesize that this taxon has a potential suppressive effect on other bacteria harmful to human health; however, under stress, it can become pathogenic.

Regarding the differences in the bacterial microbiome of the nasopharynx of infected and uninfected individuals within and between families, the results showed that each of the uninfected individuals comprises a more or less similar microbial community within their household, with discrete varying abundances of a few taxa, just as described by Abhishek Gupta et al. (2022) [37], further corroborating the idea that individuals who reside together and/or share common ecological niches contain similar microbial communities that can be subsequently shaped by factors intrinsic to the individual, diet, lifestyle, work occupation, etc. [51,52].

Alternatively, we also looked into how the nasopharyngeal microbiome varies depending on the age and sex of the individuals. What we found was that, while infected adults have similar niche characteristics, there were higher levels of certain taxa in the nasopharynx of younger adults compared to middle-aged adults. We also observed an increase in opportunistic pathogens (*Shigella sonnei*, *Klebsiella pneumoniae*, and *Staphylococcus* spp.) in the age groups of 18–31 and 32–45 in individuals infected with SARS-CoV-2. Interestingly, the microbiome of the infected nasopharynx in the 46–59 age group seemed to be the least affected, with no differences in microbial abundance when compared to the negative group. These findings support the conclusions of Abhishek Gupta et al. (2022), who suggested that microbial groups are linked to different age groups and have varying responses to SARS-CoV-2 infection [37].

Despite observing an increase in the point abundance of a few bacterial groups found in infected male or female individuals, supporting the hypothesis that they may respond differently during SARS-CoV-2 infection [11,49], our results are in line with previous studies carried out on COVID-19 patients, where no significant association of age and sex was found [37,46,49].

Furthermore, we did not detect other respiratory viruses as co-pathogens in COVID-19 patients. A plausible hypothesis for why no co-infection of SARS-CoV-2 with other respiratory viruses was detected could be related to reduced opportunities for transmission, since, during 2021, strict lockdowns, travel restrictions, and widespread use of masks likely led to a drastic reduction in the circulation of other respiratory viruses, including the ones we investigated (FLU, AdV, HBoV, HCoV, HMPV, RSV, PIV, and HRV) [53,54]. This potentially reduced the chances of finding other respiratory pathogens, leading to fewer potential co-infections at that time.

Our work also has some limitations. Unfortunately, due to the emergency public health situation at the time, it was not feasible to follow up patients and collect serial nasopharyngeal swabs from these individuals. In addition, due to the heterogeneity of our samples (age, disproportionate number of individuals—positive and negative) and lack of clinical and sociodemographic information, we were limited to establishing associations between the dysbiosis events of the nasopharyngeal microbiome found in our study and the individual characteristics of the study participants.

## 5. Conclusions

Overall, this study describes for the first time the nasopharyngeal microbiome of SARS-CoV-2-infected and uninfected individuals from a Brazilian Amazonian community. Our results suggest that SARS-CoV-2 infection has facilitated the abundance of opportunistic pathogens, unbalancing the resident commensal microbiome due to the viral infection. We also observed potential bacterial groups (*Geobacillius thermoleovorans* and *Bacteroides* spp.) with antagonistic effects to opportunistic pathogens (*Pseudomonas aeruginosa* and *Escherichia coli*) that could serve as prognostic biomarkers of the nasopharyngeal microbiome of patients with COVID-19. These biomarkers could help in the screening of high-risk patients, allow for more personalized treatment, and help in the proper allocation of medical resources. In addition, the identification of biomarkers can contribute to the development of new therapies and interventions to improve patient prognosis.

## Figures and Tables

**Table 1:** Summary of variables analyzed.

	No. Samples
**Variables**	n = 63
Individuals infected with SARS-CoV-2	n = 37
Individuals not infected with SARS-CoV-2	n = 26
Male	n = 29
Infected	n = 20
Not infected	n = 9
Female	n = 34
Infected	n = 17
Not infected	n = 17
Total Residences	n = 21
Age group 1 (18 to 31 years)	n = 26
Age group 2 (32 to 45 years)	n = 21
Age group 3 (46 to 59 years)	n = 9
Age group 4 (≥60 yeas)	n = 7

## Data Availability

The raw data supporting the conclusions of this article will be made available by the authors on request.

## Supplementary Materials

The following supporting information can be downloaded at: https://www.mdpi.com/article/10.3390/microorganisms13051088/s1, Table S1: Information on positive and negative samples for SARS-CoV-2.; Table S2: List of all bacterial families and their respective reading numbers for each study sample.
