# Supplementary material for: Impact of SARS-CoV-2 P.1 Variant Infection on the Nasopharyngeal Commensal Bacterial Microbiome of Individuals from the Brazilian Amazon
Source: Microorganisms. 2025 May 8;13(5):1088. doi: 10.3390/microorganisms13051088 (PMC12113811; doi:10.3390/microorganisms13051088)
Supplement: Supplementary file 1 [file microorganisms-13-01088-s001.zip › microorganisms-3370063-supplementary/Supplementary Material Table S1.pdf]

Supplementary Material Table S1. Information on positive and negative samples for SARS-CoV-2.

Sample no	Internal code	Age	Gender	Residence code	Real-time PCR results for SARS-CoV-2	Real-time PCR results for other respiratory viruses
1	P_178796_C1	48	Female	F01	Positive	Negative
2	P_180692_CT1.2	27	Male	F01	Positive	Negative
3	P_178812_C2	46	Male	F02	Positive	Negative
4	P_178813_CT2.1	52	Female	F02	Positive	Negative
5	P_178778_CT3.5	20	Male	F03	Positive	Negative
6	P_178792_CT3.4	32	Male	F03	Positive	Negative
7	N_180695_CT5.2	44	Male	F05	Negative	Negative
8	N_180702_CT5.3	32	Female	F05	Negative	Negative
9	P_178801_C5	41	Male	F05	Positive	Negative
10	N_180711_CT6.3	72	Female	F06	Negative	Negative
11	N_180717_CT6.4	22	Female	F06	Negative	Negative
12	N_180718_C6	25	Female	F06	Negative	Negative
13	N_180696_CT8.3	28	Female	F08	Negative	Negative
14	N_180701_CT8.2	41	Female	F08	Negative	Negative
15	P_178789_C8	63	Female	F08	Positive	Negative
16	P_178810_C9	43	Male	F09	Positive	Negative
17	N_180719_CT10.2	37	Male	F10	Negative	Negative
18	P_178787_C10	75	Male	F10	Positive	Negative
19	P_180683_CT10.1	44	Male	F10	Positive	Negative
20	N_180697_CT11.1	22	Female	F11	Negative	Negative
21	N_180682_CT12.7	43	Male	F12	Negative	Negative
22	N_180705_CT12.8	20	Female	F12	Negative	Negative
23	N_180715_CT12.4	21	Male	F12	Negative	Negative
24	N_180720_CT12.2	26	Male	F12	Negative	Negative
25	N_180721_CT12.3	42	Female	F12	Negative	Negative
26	N_180722_CT12.5	37	Male	F12	Negative	Negative
27	N_180725_CT12.6	32	Female	F12	Negative	Negative
28	P_180690_C12	53	Female	F12	Positive	Negative
29	N_180723_CT13.2	22	Female	F13	Negative	Negative
30	P_180688_C13	73	Female	F13	Positive	Negative
31	N_180724_C14	26	Female	F14	Negative	Negative
32	P_178807_CT14.2	56	Female	F14	Positive	Negative
33	N_180698_CT15.6	20	Male	F15	Negative	Negative
34	N_180700_CT15.4	19	Female	F15	Negative	Negative
35	N_180713_CT15.5	60	Female	F15	Negative	Negative
36	P_178779_C15	38	Male	F15	Positive	Negative
37	P_178780_CT15.1	23	Female	F15	Positive	Negative
38	P_178781_CT15.2	33	Male	F15	Positive	Negative
39	P_178782_CT15.3	26	Female	F15	Positive	Negative
40	P_178786_CT15.7	21	Female	F15	Positive	Negative
41	P_178809_C16	29	Male	F16	Positive	Negative
42	P_180689_CT16.2	27	Female	F16	Positive	Negative
43	P_178783_C17	39	Female	F17	Positive	Negative
44	P_178784_CT17.1	48	Male	F17	Positive	Negative
45	P_178785_CT17.2	21	Male	F17	Positive	Negative
46	N_180714_CT19.2	19	Female	F19	Negative	Negative
47	P_178788_C19	60	Male	F19	Positive	Negative
48	P_178790_CT19.1	55	Female	F19	Positive	Negative
49	P_178797_C20	26	Female	F20	Positive	Negative
50	P_178798_CT20.1	24	Male	F20	Positive	Negative
51	P_178799_CT20.2	37	Male	F20	Positive	Negative
52	N_180709_CT21.1	36	Female	F21	Negative	Negative
53	P_178814_C21	41	Male	F21	Positive	Negative
54	N_180694_CT22.1	50	Female	F22	Negative	Negative
55	N_180699_CT22.4	27	Male	F22	Negative	Negative
56	P_178795_C22	28	Male	F22	Positive	Negative
57	P_180691_CT22.5	46	Male	F22	Positive	Negative
58	N_180707_CT23.2	26	Male	F23	Negative	Negative
59	P_178800_C23	26	Female	F23	Positive	Negative
60	P_178802_CT23.1	64	Female	F23	Positive	Negative
61	P_178804_CT23.4	44	Female	F23	Positive	Negative
62	P_178794_CT24.1	42	Female	F24	Positive	Negative
63	P_180684_C24	43	Male	F24	Positive	Negative

Other respiratory viruses: Influenza A virus (FluA), Influenza B virus (FluB), Adenovírus (AdV), Bocavírus humano (HBoV), Coronavírus humano (HCoV) 229E, HKU1, NL63 e OC43; Metapenumovírus humano (HMPV), Orthopneumovírus humano (HOPV), Respirovírus humanos 1 e 3 (PIV 1 e 3), Rinovírus humano (HRV) e Rubulavírus humano 2 (PIV 2).
