# Supplementary material for: Impact of SARS-CoV-2 P.1 Variant Infection on the Nasopharyngeal Commensal Bacterial Microbiome of Individuals from the Brazilian Amazon
Source: Microorganisms. 2025 May 8;13(5):1088. doi: 10.3390/microorganisms13051088 (PMC12113811; doi:10.3390/microorganisms13051088)
Supplement: Supplementary file 1 [file microorganisms-13-01088-s001.zip › microorganisms-3370063-supplementary/Supplementary Material Table S2.pdf]

Supplementary Material Table S2. List of all bacterial families and their respective reading numbers for each study sample.

	No. Reds		
Family	Positive samples (%)	Negative samples (%)	Positive + Negative samples (%)
Enterobacteriaceae	15103442 (57,52)	5575372 (26,96)	20678871 (48,45)
Bacillaceae	4259293 (58,12)	1534314 (26,48)	5793665 (13,57)
Vibrionaceae	2934472 (56,24)	1141483 (28,00)	4076011 (9,55)
Lactobacillaceae	1236415 (53,28)	541931 (30,47)	1778399 (4,16)
Bacteroidaceae	941767 (57,75)	344448 (26,77)	1286272 (3,014)
Staphylococcaceae	849134 (50,22)	420773 (33,13)	1269957 (2,97)
Xanthomonadaceae	768791 (52,97)	341184 (30,73)	1110027 (2,60)
Borreliaceae	477700 (86,11)	38515 (7,45)	516301 (1,20)
Nostocaceae	410010 (60,81)	132079 (24,36)	542149 (1,27)
Streptomycetaceae	351631 (87,19)	25823 (6,83)	377541 (0,88)
Burkholderiaceae	315892 (59,16)	109029 (25,65)	424980 (0,99)
Erwiniaceae	193695 (72,15)	37380 (16,17)	231147 (0,54)
Pseudomonadaceae	355081 (20,58)	684749 (65,85)	1039850 (2,43)
Listeriaceae	111873 (54,41)	46852 (29,50)	158779 (0,37)
Morganellaceae	142096 (64,77)	38631 (21,36)	180791 (0,42)
Campylobacteraceae	42549 (52,87)	18963 (30,80)	61564 (0,14)
Moraxellaceae	78788 (32,89)	80355 (50,48)	159175 (0,37)
Clostridiaceae	52561 (32,57)	54395 (50,84)	106988 (0,25)
Enterococcaceae	102728 (26,95)	139218 (57,53)	241972 (0,56)
Streptococcaceae	112270 (25,09)	167597 (59,87)	279892 (0,65)
Yersiniaceae	23729 (34,82)	22202 (48,30)	45965 (0,10)
Propionibacteriaceae	75893 (21,25)	140617 (64,94)	216531 (0,50)
Lachnospiraceae	30231 (26,03)	42952 (58,67)	73209 (0,17)
Alcaligenaceae	12749 (33,07)	12897 (50,22)	25679 (0,06)
Hydrogenophilaceae	27920 (21,57)	50746 (64,49)	78687 (0,18)
Peptostreptococcaceae	28923 (25,10)	43150 (59,84)	72098 (0,16)
Corynebacteriaceae	137164 (20,84)	260402 (65,49)	397586 (0,93)
Mycobacteriaceae	48113 (30,78)	54089 (52,90)	102232 (0,23)
Barnesiellaceae	334 (10,65)	1401 (80,25)	1745 (0,00)
Muribaculaceae	70 (100)	0 (0)	170 (0,00)
Paludibacteraceae	58 (100)	0 (0)	158 (0,00)
Porphyromonadaceae	10667 (98,50)	81 (0,74)	10846 (0,025)
Prevotellaceae	27453 (56,38)	10617 (27,84)	38126 (0,08)
Rikenellaceae	2509 (26,66)	3450 (57,63)	5985 (0,01)
Tannerellaceae	11578 (26,25)	16263 (58,35)	27867 (0,06)
Marinifilaceae	5137 (26,14)	7255 (58,42)	12418 (0,02)
Chitinophagaceae	0 (0)	185 (100)	185 (0,00)
Cyclobacteriaceae	229 (100)	0 (0)	329 (0,00)
Cytophagaceae	124 (100)	0 (0)	224 (0,00)
Flavobacteriaceae	37664 (22,72)	64041 (62,95)	101727 (0,23)
Weeksellaceae	1942 (31,63)	2098 (51,52)	4071 (0,00)
Sphingobacteriaceae	6197 (22,13)	10899 (63,66)	17118 (0,04)
Fusobacteriaceae	3607 (97,61)	44 (1,17)	3748 (0,00)
Leptotrichiaceae	0 (0)	83 (100)	83 (0,00)
Acidithiobacillaceae	40328 (22,79)	68292 (62,85)	108642 (0,25)
Caulobacteraceae	51 (2,11)	1181 (95,69)	1234 (0,00)
Bradyrhizobiaceae	776 (24,80)	1176 (59,48)	1976 (0,00)
Brucellaceae	952 (57,55)	351 (25,79)	1360 (0,00)
Methylobacteriaceae	765 (26,59)	1056 (57,15)	1847 (0,00)
Phyllobacteriaceae	140 (10,40)	603 (80,03)	753 (0,00)
Rhizobiaceae	8426 (28,75)	10440 (55,25)	18894 (0,04)
Stappiaceae	114 (9,77)	526 (80,95)	649 (0,00)
Rhodobacteraceae	4150 (11,90)	15360 (78,68)	19521 (0,04)
Roseobacteraceae	0 (0)	353 (100)	353 (0,00)
Acetobacteraceae	675 (16,31)	1731 (71,46)	2422 (0,00)
Thalassospiraceae	0 (0)	44 (100)	44 (0,00)
Erythrobacteraceae	71 (52,59)	32 (20,56)	156 (0,00)
Sphingomonadaceae	2221 (21,75)	3994 (64,03)	6237 (0,01)
Sphingosinicellaceae	47 (100)	0 (0)	147 (0,00)
Comamonadaceae	2051 (12,70)	7049 (77,35)	9113 (0,02)
Oxalobacteraceae	2857 (27,45)	3774 (56,67)	6658 (0,01)
Chromobacteriaceae	24 (8,88)	123 (78,90)	156 (0,00)
Neisseriaceae	289 (1,82)	7774 (96,39)	8065 (0,01)
Rhodocyclaceae	27 (12,32)	96 (70,93)	135 (0,00)
Zoogloeaceae	0 (0)	92 (100)	92 (0,00)

Continue. Supplementary Material Table 2. List of all bacterial families and their respective reading numbers for each study sample.

	No. Reds		
Family	Positive samples (%)	Negative samples (%)	Positive + Negative samples (%)
Desulfovibrionaceae	1868 (27,04)	2520 (57,07)	4415 (0,01)
Myxococcaceae	432 (15,98)	1135 (71,69)	1583 (0,00)
Helicobacteraceae	955 (28,09)	1222 (55,41)	2205 (0,00)
Aeromonadaceae	191 (22,08)	337 (61,26)	550 (0,00)
Alteromonadaceae	877 (48,64)	463 (33,34)	1389 (0,00)
Colwelliaceae	1370 (28,72)	1700 (54,86)	3099 (0,00)
Idiomarinaceae	0 (0)	21 (100)	21 (4,92)
Pseudoalteromonadaceae	10171 (23,79)	16287 (61,50)	26482 (0,06)
Psychromonadaceae	729 (7,36)	4587 (86,16)	5323 (0,01)
Shewanellaceae	75 (13,56)	239 (72,96)	328 (0,00)
Chromatiaceae	946 (100)	0 (0)	1046 (0,00)
Pectobacteriaceae	1688 (4,99)	16067 (90,46)	17760 (0,04)
Legionellaceae	375 (26,61)	517 (56,28)	919 (0,00)
Halomonadaceae	293 (56,89)	111 (24,083)	461 (0,00)
Oceanospirillaceae	290 (22,87)	489 (60,98)	802 (0,00)
Pasteurellaceae	12070 (33,54)	11956 (49,69)	24060 (0,05)
Francisellaceae	2277 (33,56)	2253 (49,36)	4564 (0,01)
Chlamydiaceae	1221 (92,57)	49 (3,59)	1363 (0,00)
Leptospiraceae	608 (26,97)	823 (56,44)	1458 (0,00)
Treponemataceae	3726 (100)	0 (0)	3826 (0,00)
Synergistaceae	308 (19,56)	633 (65,89)	961 (0,00)
Actinomycetaceae	1150 (24,75)	1748 (59,80)	2923 (0,00)
Bifidobacteriaceae	61256 (24,21)	95868 (61,00)	157148 (0,36)
Dietziaceae	0 (0)	296 (100)	296 (0,00)
Gordoniaceae	133 (17,52)	313 (67,52)	464 (0,00)
Lawsonellaceae	8434 (28,46)	10597 (55,59)	19059 (0,04)
Nocardiaceae	371 (8,03)	2123 (84,85)	2502 (0,00)
Tsukamurellaceae	0 (0)	37 (100)	37 (8,67)
Frankiaceae	206 (100)	0 (0)	306 (0,00)
Brevibacteriaceae	506 (17,72)	1174 (69,15)	1698 (0,00)
Cellulomonadaceae	579 (100)	0 (0)	679 (0,00)
Dermabacteraceae	99 (6,87)	671 (86,37)	777,87 (0,00)
Dermacoccaceae	596 (11,66)	2256 (78,78)	2864 (0,00)
Intrasporangiaceae	3694 (33,02)	3746 (50,12)	7473 (0,01)
Kytococcaceae	115 (25,27)	170 (54,79)	310 (0,00)
Microbacteriaceae	1069 (16,85)	2636 (70,82)	3722 (0,00)
Micrococcaceae	59102 (10,49)	252060 (81,00)	311172 (0,72)
Ornithinimicrobiaceae	73 (100)	0 (0)	173 (0,00)
Micromonosporaceae	0 (0)	41 (100)	41 (9,60)
Nocardioidaceae	337 (18,40)	747 (67,76)	1102 (0,00)
Pseudonocardiaceae	645 (19,59)	1323 (66,56)	1988 (0,00)
Nocardiopsaceae	0 (0)	343 (100)	343 (0,00)
Thermomonosporaceae	1892 (16,69)	4722 (71,21)	6631 (0,01)
Eggerthellaceae	1429 (26,78)	1953 (57,29)	3409 (0,00)
Rubrobacteraceae	811 (26,46)	1127 (57,36)	1964 (0,00)
Chroococcidiopsidaceae	217 (35,51)	197 (43,82)	450 (0,00)
Aphanizomenonaceae	226 (100)	0 (0)	326 (0,00)
Hapalosiphonaceae	571 (100)	0 (0)	671 (0,00)
Scytonemataceae	225 (22,12)	396 (61,57)	643,12 (0,00)
Chroococcaceae	194 (100)	0 (0)	294 (0,00)
Microcoleaceae	135 (100)	0 (0)	235 (0,00)
Leptolyngbyaceae	179 (100)	0 (0)	279 (0,00)
Deinococcaceae	6476 (18,53)	14228 (68,65)	20723 (0,04)
Alicyclobacillaceae	0 (0)	55 (100)	55 (0,00)
Paenibacillaceae	58814 (19,86)	118594 (66,84)	177428 (0,41)
Planococcaceae	75 (8,98)	380 (81,89)	464 (0,00)
Carnobacteriaceae	2987 (41,27)	2125 (41,23)	5153 (0,01)
Eubacteriaceae	861 (35,77)	773 (46,29)	1670 (0,00)
Eubacteriales Family XIII	153 (100)	0 (0)	253 (0,00)
Oscillospiraceae	3115 (21,76)	5600 (64,096)	8737 (0,020)
Peptococcaceae	535 (26,89)	727 (56,40)	1289 (0,00)
Thermoanaerobacteraceae	0 (0)	37 (100)	37 (8,67)
Thermoanaerobacterales Family III	36 (9,47)	172 (79,09)	217 (0,00)
Erysipelotrichaceae	12821 (18,62)	28012 (68,57)	40852 (0,09)
Acidaminococcaceae	163 (15,33)	450 (71,61)	628 (0,00)

Continue. Supplementary Material Table 2. List of all bacterial families and their respective reading numbers for each study sample.

Family	Positive samples (%)	Negative samples (%)	Positive + Negative samples (%)
Selenomonadaceae	84 (35,59)	76 (38,85)	196 (0,00)
Sporomusaceae	3687 (22,37)	6395 (63,28)	10104 (0,02)
Veillonellaceae	7169 (29,56)	8538 (54,25)	15737 (0,03)
Peptoniphilaceae	12046 (52,21)	5511 (31,29)	17609 (0,04)
Tissierellaceae	165 (27,63)	216 (52,85)	409 (0,00)
Mycoplasmataceae	10277 (16,13)	26706 (72,18)	36999 (0,08)
Total reads	29700621 (69,60)	13005158 (30,48)	42673746
